# *ATG*8 and protein ATG8ylation – more than just *A*nother *T*a*G*?

**DOI:** 10.1080/15548627.2026.2642981

**Published:** 2026-03-20

**Authors:** Fabian Gerth, Simone Kosol, Natasha Aley, Alexander Agrotis, Robin Ketteler

**Affiliations:** a Department of Human Medicine, Medical School Berlin, Berlin, Germany; b UCL Cancer Institute, University College London, London, UK; c Research Department of Cell and Developmental Biology, Division of Biosciences, University College London, London, UK

**Keywords:** ATG4B, ATG8, ATG8ylation, autophagy, LC3ylation, ubiquitin-like

## Abstract

Since its discovery as a key component of the autophagosome membrane, the small ubiquitin-like protein ATG8 and its mammalian homologs (ATG8s) have garnered a lot of attention. Many researchers use it as a marker for autophagosome number, size and composition. A lot of research has focussed on its function in forming complexes required for autophagosome-lysosome fusion or generally, its interaction with other proteins via the ATG8-family interacting motif/AIM. Many additional functions have been discovered, for instance in non-canonical autophagy processes and in the nucleus. The list of known functions of ATG8 are ever expanding, and, most recently, evidence has emerged that, similar to ubiquitin, ATG8 can modify proteins by covalent attachment to a lysine residue (protein ATG8ylation). In this review, we aim to summarize the current literature on protein ATG8ylation and highlight the currently known substrates. We propose strategies to investigate this modification and provide an outlook for its possible cellular function.

**Abbreviations**: ATG: autophagy related; DUBs: de-ubiquitinating enzymes; GABARAPL: GABA type A receptor associated protein like; GIR: GABARAP-interacting region; LIR: LC3-interacting region; MAP1LC3: microtubule associated protein 1 light chain 3; RMSD: root mean square; UBL: ubiquitin-like; UPS: ubiquitin-proteasome-system.

## Introduction

**Figure 1.**
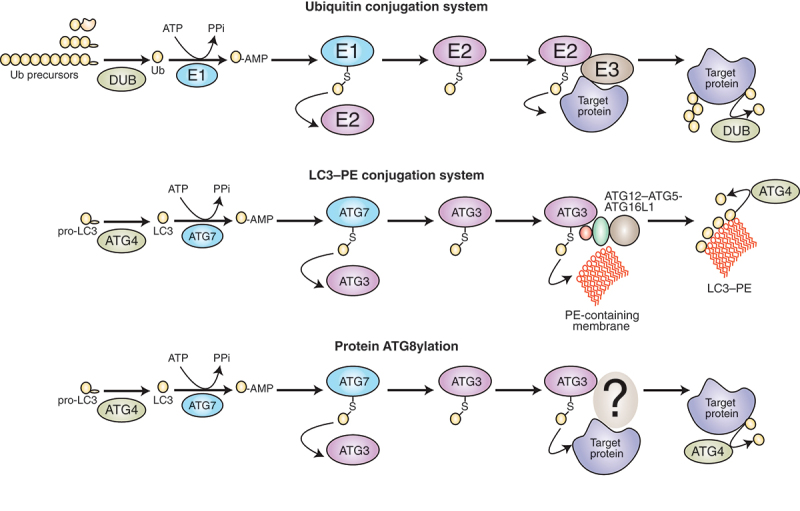
Comparison of similarities of ubiquitination and ATG8ylation. In the ubiquitin-proteasome-system, ubiquitin precursors are first cleaved by de-ubiquitinating enzymes (DUBs) to produce ubiquitin. Ubiquitin is then tagged to lysine residues of proteins via the concerted action of E1, E2 and E3 enzymes. DUBs are also capable of removing ubiquitin from proteins, e.g., prior to entry into the proteasome channel. In analogy, pro-ATG8 is first cleaved by ATG4 proteases and subsequently conjugated to lipid membranes via the concerted action of E1-, E2- and E3-like enzymes (ATG7, ATG3, ATG5 complex). Protein ATG8ylation is also mediated by ATG7 and ATG3, and an E3-like enzyme has not yet been identified.

The two main degradation pathways in cells are the ubiquitin-proteasome system (UPS) and the macroautophagy/autophagy-lysosome system [1,2]. There are many commonalities between these two systems (Figure 1) [3]. Both pathways involve ubiquitin or ubiquitin-like proteins to mark the substrates for degradation [4,5] and a set of E1-, E2- and E3-like enzymes to facilitate the tagging of its substrate for degradation [6]. In the case of proteasome-mediated degradation, the tagging of substrates with ubiquitin is mediated in three steps in an ATP-dependent manner by the activating (E1) and conjugating (E2) enzymes and the E3 ubiquitin ligase. In the case of autophagy, a similar set of E1, E2 and E3-like conjugation enzymes exist, that facilitate the processing of the mammalian ATG8-like proteins MAP1LC3/GABARAP (MAP1LC3A, MAP1LC3B, MAP1LC3B2, MAP1LC3C, GABARAP, GABARAPL1, GABARAPL2) [6–9]. First, pro-MAP1LC3/GABARAP is cleaved at the C terminus by members of the ATG4 family [10]. This exposes a glycine residue that is conjugated to either phosphatidylethanolamine or phosphatidylserine in the phagophore membrane through the concerted action of the E1- and E2-like enzymes ATG7 and ATG3 and the E3-like complex of ATG5, ATG12, and ATG16L1 in an ATP-consuming reaction [11]. In the UPS system, ubiquitin is removed by de-ubiquitinating enzymes (DUBs) prior to entry in the proteasomal channel. Similar DUB-like enzymes have also been described operating in the autophagy-lysosome system. The DUB-like enzymes operating in autophagy are members of the ATG4 family of proteins, though they may have roles in addition to recycling of MAP1LC3/GABARAP [12–16]. ATG4 proteins can thus be referred to as “De-ATG8ylases.”

**Figure 2.**
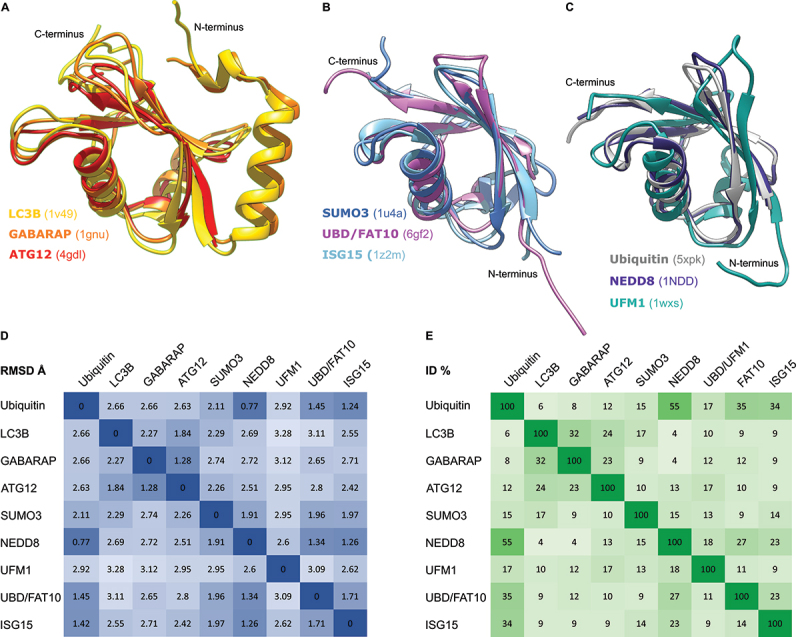
Structural comparison of ubiquitin-like proteins (UBLs). (A) Superimposition of the autophagy-related UBLs from the ATG8 family, MAP1LC3B (yellow), GABARAP (orange), and ATG12 (red; IDR not shown). (B) Superimposition of UBLs involved in stress-response, immune-response and immunomodulation: SUMO3 (blue), UBD/FAT10 (lilac), and ISG15 (light blue). (C) Superimposition of ubiquitin and UBLs involved in protein degradation und quality control: ubiquitin (grey), NEDD8 (purple), UFM1 (cyan). (D) Similarity matrix based on pairwise structural alignments of UBLs using the sequence-independent protein structure comparison method TM-align [17] in the RCSB protein data bank tool [18]. Root mean square deviation (RMSD) values are represented by colour intensity, with darker hues indicating higher structural similarity. The RMSD is calculated in Å between aligned pairs of the backbone C-alpha atoms. (E) Sequence identity matrix based on sequence identities in the structural alignments represented by color intensity, with darker hues indicating higher structural similarity. The sequence identity is calculated as the percent of paired residues that are identical matches in the alignment.

Although ATG8 family members and ATG12 share only limited sequence similarity with ubiquitin, they all adopt the characteristic β-grasp fold that is conserved among ubiquitin-like (UBL) proteins (Figure 2) [19]. Typically, the members of the ATG8 family have two N-terminal α-helices extending from the ubiquitin-like core [20], which play a role in binding multiple proteins carrying the MAP1LC3/LC3-interacting region (LIR) motif [21]. ATG12, on the other hand, possesses an intrinsically disordered N-terminal region (due to its flexibility not present in the X-ray structure) which is essential for its function in yeast [22]. Flexible N-terminal extensions have also been described for members of the SUMO family [23]. A recent study suggested that the intrinsically disordered N-terminus of SUMO1 acts as intramolecular inhibitor by blocking interactions between SUMO1 and its targets or downstream effector proteins [24]. Alignments of the three autophagy-related proteins MAP1LC3B, GABARAP and ATG12 suggest high structural similarity in their UBL-cores with root mean square (RMSD) values between structures ranging from 1.28 to 2.27 Å. The RMSD quantifies the average distance between the C-alpha atoms of the aligned structures, with a lower RMSD indicating higher similarity. The sequence similarity among the ATG8 family members is moderate (23–32% identity in pairwise aligned structures) but higher than to other UBLs (<20% identity). Notably, MAP1LC3B, GABARAP, and ATG12 are more structurally similar to one another than to other UBLs or to ubiquitin itself (Figure 2), although the β-grasp structure is clearly well-conserved across all. In light of reports describing a shared interaction motif of the E1-enzyme UBA5 (ubiquitin like modifier activating enzyme 5) for both UFM1 and MAP1LC3/GABARAP [25], the relatively low structural similarity between UFM1 and the ATG8 family members – compared to other UBLs – is somewhat surprising.

### The many faces of ATG8

Genetic screens for autophagy-defective mutants in yeast in the early 1990s identified a set of *APG* and *AUT* genes essential for autophagosome formation [26]. Among these was a gene originally designated YBL078C, also named Apg8, Aut7, and later standardized as ATG8 in the unified autophagy nomenclature [27,28]. ATG8 encodes a ubiquitin-like protein that is conjugated to the membrane lipid phosphatidylethanolamine (PE) and is required for expansion and closure of phagophore membranes during autophagy [29,30]. Its essential role in both bulk autophagy and the cytoplasm-to-vacuole targeting/Cvt pathway established a core mechanistic insight into this conserved degradative pathway.

Originally characterized as components of microtubule-associated proteins MAP1A and MAP1B [31,32], the mammalian ATG8 family of proteins has now been implicated in multiple processes. MAP1LC3B is the most studied and understood of the ATG8 family. Transcription of ATG8 proteins has been shown to be controlled by a variety of environmental responses and transcription factors including GATA1 [33], FOXO3 [33], FOXO1 [34,35], SREBF2 (sterol regulatory element binding transcription factor 2), CEBP (CCAAT enhancer binding protein) [36] and JUN/c-jun [37,38]. Binding sites within the MAP1LC3B promoter for the transcription factor TFEB have been identified by ChIP-seq [39]. Interestingly, ATG8 regulates the activity of TFEB-mediated transcription by interaction with IRGM [40], pointing to a complex role of ATG8 family members in the transcriptional regulation of autophagy. At the post-translational level, the ATG8 proteins are regulated by phosphorylation [41–43], acetylation [44], ubiquitination [45] and redox mechanisms [46–48]. MAP1LC3B is commonly used as a marker for autophagy and is a potential biomarker for various diseases including cancer [49].

The main functions of ATG8 can be summarized in four main categories: 1) autophagosome formation and closure; 2) lysosome fusion; 3) selective autophagy receptor binding; and 4) conjugation of ATG8 to single membranes (CASM) such as in LC3-associated phagocytosis/LAP [50], LC3-associated endocytosis/LANDO [51], LC3C-endosome associated pathway/LEAP [52] and LC3-dependent exosomal loading and secretion/LDELS [53]. For a detailed overview on these functions, please refer to other published reviews [54–58]. Lipidation-independent roles were suggested for cytosolic ATG8 proteins such as a function in vacuolar morphology maintenance in yeast and ER-associated degradation/ERAD in mammalian cells [59,60].

In *S. cerevisiae*, deletion of *ATG8* abolishes autophagosome formation [27] and deletion of *ATG3*, encoding a key protein in the lipidation of ATG8, results in malformed autophagosomes [61]. These findings led to the suggestion that proteins of the ATG8 family are essential for autophagosome biogenesis. However, CRISPR-Cas9 knockout (KO) of six of the seven genes encoding ATG8 protein family members in HeLa cells did not abolish the formation and nucleation of autophagosomes, but instead led to defects in size and efficiency of autophagosome formation and lysosomal fusion [62]. It is unlikely that the residual MAP1LC3B2 accounts for this effect since it is expressed at very low levels [63] and was not detectable by western blotting in the cells in this study. In this knockout model, it was, however, observed that the loss of MAP1LC3 and GABARAP family members prevented the recruitment of PLEKHM1 to the autophagosome membrane, a factor required for autophagosome-lysosome fusion [64,65].

Blocking ATG8 conjugation by knockout of *ATG3*, *ATG5* or *ATG7* results in a delayed degradation of the inner autophagosome membrane, rather than a complete failure of autophagosome-lysosome fusion [66]. This highlights that ATG8 proteins could have additional functions independent of conjugation. The same paper also demonstrated that ATG3 played a role in autophagosome fusion, and that knockout of *ATG3* prevents the closure of some phagophores [66]. Interestingly, the N-terminal amphipathic helix of ATG3 has been noted to sense membrane curvature [67] and to enable ATG3 to associate with the highly curved tips of a growing phagophore. Furthermore, this study also demonstrated that ATG8 conjugation is membrane curvature dependent. For instance, GABARAPL1 prefers conjugation on highly curved liposomes compared to other ATG8 members.

In addition to these functions, it is possible that ATG8s serve as platforms for interaction with other proteins via binding to LIRs or GABARAP-interacting regions (GIR) [55,68–70]. Several labs have published large proteomic datasets of the MAP1LC3 interactome [71–77], demonstrating a complex network or autophagy and non-autophagy proteins interacting with ATG8 proteins. The presence of LIR and GIR domains seems quite widespread [68], thus such interaction hubs could be complex and important for cellular function.

### Membrane ATG8ylation

**Figure 3.**
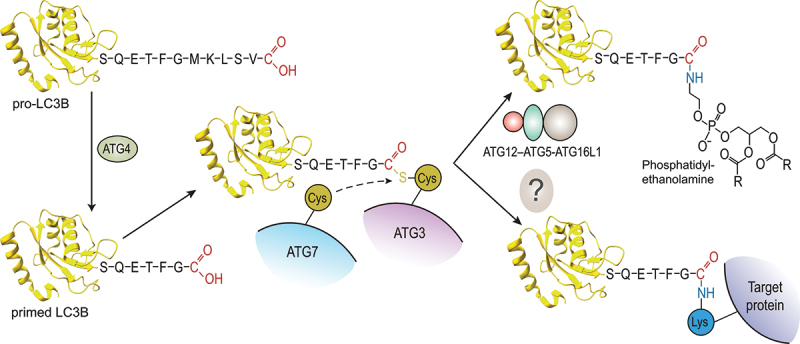
Covalent modifications of the MAP1LC3B C-terminus during membrane and protein ATG8ylation. After cleavage of the C-terminal five residues of pro-MAP1LC3B by the protease ATG4, the newly exposed glycine carboxy-terminus is activated and transferred to ATG7 where it forms a transient thioester bond with a cysteine sidechain. It is subsequently passed on to ATG3, again forming a transient thioester bond, and then finally transferred to its lipid or protein target. During membrane ATG8ylation, this final transfer is enhanced by the E3-like ATG12–ATG5-ATG16L1 complex, while the E3 for protein ATG8ylation is still unknown. Linkage of MAP1LC3B to its substrates is achieved via an amide bond between the C-terminal glycine and the lipids phosphatidylethanolamine and phosphatidylserine or the lysine side chain of a protein (e.g., K163 of ATG3).

The conjugation of ATG8 to its substrate is remarkably similar to the ubiquitin conjugation system (Figure 1). The process involves a series of consecutive steps, where the ATG8 C-terminus is processed and then transiently linked to a number of E1- and E2-like enzyme before it is finally transferred to its substrate (Figure 3). Newly synthesized ATG8 (pro-ATG8 or ATG8-I) is first rapidly primed by a cysteine protease of the ATG4 family by cleaving one or more amino acid residues found at the C-terminus exposing an active glycine residue [10,28,78]. ATG7, an E1-like enzyme, activates the carboxyl group of this glycine residue in an ATP-dependent manner to generate an acyl-adenylate intermediate. A thioester is formed with a cysteine sidechain of ATG7 enabling the subsequent transfer of ATG8 to the E2-like enzyme ATG3 to enable conjugation to the lipid by the E3-like ATG12–ATG5-ATG16L1 complex. This leads to the formation of an amide bond between the C-terminal alpha-carboxy group of ATG8 and the head moiety of a phospholipid in both the inner and outer compartment of an phagophore membrane [79]. The phospholipid conjugation is reversible, as ATG4 can act as a de-ubiquitinating-like enzyme and remove ATG8 from the membrane by delipidation [10,78]. Although all of the ATG8 proteins undergo this ubiquitin-like conjugation, there are differences in their efficiency and specificity. Analysis from cell-free assays exposing delipidated ATG8 to ATP, ATG7, ATG3 and liposomes has revealed that phospholipid conjugation is most efficient for GABARAPL1, followed by GABARAPL2 and the least efficient in MAP1LC3B [67]. Conjugation can occur in the absence of the ATG12–ATG5-ATG16L1 complex *in vitro*, but the presence of this E3-like complex greatly enhances the rate of conjugation [11,80,81]. Recruitment of the ATG12–ATG5-ATG16L1 complex is mediated by the action of phosphatidylinositol 3-kinase complex and WIPI2 [82,83] and the ULK1/ULK2 complex [84,85]. In the case of CASM, the v-type ATPase is involved in recruitment of ATG12–ATG5-ATG16L1 in response to disruption of the proton gradient across the membrane and ULK1 or ULK2 may not be required [86–88]. The conjugation reaction is also affected by membrane curvature and composition [67,89–91], as well as post-translational modifications of ATG8 and ATG4 proteins. For instance, a recent study showed that MAP1LC3C phosphorylation can prevent both its initial processing by ATG4 and subsequent conjugation and delipidation from membranes [41]. In addition, it was demonstrated that MAP1LC3 conjugation to the phagophore membrane can be suppressed by an inhibitory phosphorylation of ATG4 by ATG1 in yeast [92] and of ATG4B by ULK1 in human cells [93].

ATG4 isoforms process the ATG8 isoforms with partially overlapping redundancies [12,14]. ATG4B is the most dominant and therefore the most studied of the four mammalian isoforms of ATG4 (ATGA, B, C and D). ATG4B has been reported to be able to cleave all ATG8s^10^ and to be regulated by oxidation [46], phosphorylation [93–95], nitrosylation [96] and glycosylation [97]. ATG4C and ATG4D contain a CASP3 (caspase 3) cleavage domain and it was previously suggested that caspase cleavage was required for their full activity *in vitro* [98], however this may not be the case in cells [12]. Both ATG4C and ATG4D can cleave GABARAPL1 and GABARAPL2, with ATG4D being the most effective of the two [12,13]. ATG4A cleaves GABARAPL2 the most efficiently, followed by GABARAP and is the least efficient with MAP1LC3B [99,100]. Overexpression of a dominant negative and inactive ATG4A mutant (ATG4A^C77A^) increases the number of unfused autophagosomes [101], which is in line with the hypothesis that GABARAPL2 plays a vital role in vesicle closure [101,102].

### Protein ATG8ylation

The main substrates for ATG8 conjugation are phosphatidylethanolamine and phosphatidylserine [50,103] in the membrane. Recently, it was observed that ATG8 can conjugate to proteins as well (Figure 3, Table 1) [14,16,104]. An accumulation of MAP1LC3B-ylated bands was found by western blotting in cell lines that lack ATG4 family members expressing pre-processed MAP1LC3B, suggesting that these species accumulate when the de-conjugating enzyme (e.g., ATG4B) is missing. These MAP1LC3Bylated bands are also observed for a MAP1LC3B^Q116P^ mutant that is de-conjugation resistant [16]. Interestingly, protein ATG8ylation can occur as mono-ATG8ylation or poly-ATG8ylation in chains similar to those observed for ubiquitination [105].

**Table 1.** Known ATG8ylated proteins.

Substrate	Linkage Site	Cleavable	Reference
ATG3	K163	ATG4B	[1]
ATG7	K140	ATG4B	[2,3]
ATG16L1	Unknown	ATG4B	[4]
NUFIP2	Unknown	?	[5]

To date, only a few substrates have been identified. These can be separated in two categories: a) proteins of the ATG8ylation machinery such as ATG3, ATG7 and ATG16L1, and b) other, non-autophagy related proteins such as NUFIP2. The latter supports the notion that protein ATG8ylation may have roles in general protein function rather than be limited to regulating the conjugation of ATG8s. Recently, a proteomic study identified a large range of putative additional substrates for protein ATG8ylation, many of which include non-autophagy genes [105]. Further studies are required to validate the ATG8ylation of these genes and its functional relevance.

To date, the best characterized targets of protein ATG8ylation are members of the ATG8ylation machinery. One substrate that was identified is ATG3 [16]. Mapping of the potential linkage site revealed that the MAP1LC3Bylation occurs between the C-terminal carboxy-group of the glycine in primed MAP1LC3B and lysine K243 in ATG3 (Figure 3). Another substrate is ATG7 [104,106], which is conjugated at lysine K140 [105]. A recent study identified ATG16L1 [14] as a substrate for ATG8ylation, but the linkage site for ATG8 is not known. Similar to ATG8ylation of ATG3, ATG8ylation of ATG16L1 did not change in response to autophagy modulation, suggesting an autophagy-independent role of this process. To date, one ATG8ylated protein that is not part of the autophagy pathway has been identified, NUFIP2 [107], suggesting that ATG8ylation is a more widespread phenomenon in cells.

Interestingly, all ATG8 isoforms can attach to proteins, as revealed by combination knockouts of the four ATG4 isoforms [16]. ATG8ylation is not enhanced or induced by torin 1 or bafilomycin A_1_ treatment, suggesting autophagy-independent mechanisms, though a more detailed analysis is required.

Using *ATG3* and *ATG7* knockout cells, it was shown that protein MAP1LC3Bylation requires the ATG3 and ATG7 proteins [104,105]. However, the putative E3 ligase, if any, is currently unknown and does not involve ATG5, because protein MAP1LC3Bylation can be observed in ATG5-deficient cells at similar levels as in wild-type cells [104]. There is evidence that an E3-like conjugation complex for protein MAP1LC3Bylation exists in cells based on the observation that protein ATG8ylation does not occur in *E. coli* reconstituted with ATG3 and ATG7 alone [105].

## Outlook: what, why and how are proteins ATG8ylated?

To date, very few ATG8ylated proteins have been identified. Efforts to increase the knowledge of the number of ATG8ylated proteins are underway. Some clues might be learned from novel tools available to study protein ATG8ylation or from proteomics data from ATG8-affinity isolation or ATG8 proximity labeling experiments. Both should include not only novel interaction partners, but also proteins that are covalently linked to ATG8 family members. Recently, a large number of putative targets of protein ATG8ylation has been identified by proteomics, requiring further validation [105]. The two steps in ATG8 processing, pro-ATG8 cleavage and de-lipidation, both cleave amide bonds, although the residues and composition surrounding the bond is very different. It may be possible to explore these differences to design strategies in order to specifically identify substrates that are linked by one or the other. The pro-peptide cleavage and the proteolipid cleavage by the ATG4 proteins occur with different kinetics [13]. It has been observed that the peptide cleavage is fast and complete, whereas the lipid cleavage occurs at a much slower rate and is potentially regulated either by abundance of ATG4 or by post-translational mechanisms.

Interestingly, Legionella bacteria have evolved to deploy a variant of ATG4, RavZ, that cleaves lipidated ATG8 of their host cells with a high efficiency and rapid kinetics, but does not cleave the peptide bond in pro-ATG8 or in other protein contexts [108]. It is thought that this is in part mediated by differences in the LIR domains of RavZ compared to mammalian ATG4 [109]. This poses the question whether RavZ is also incapable of cleaving the linkage in ATG8ylated proteins. If so, RavZ could be a tool to remove lipid-anchored ATG8 from membranes, while ATG8ylation on proteins remains intact.

Interestingly, ATG8 isoforms with a C-terminally exposed glycine exist in some organisms, circumventing the priming step that is usually mediated by ATG4 proteins. For instance, plants have several ATG8 isoforms, one of which (ATG8i) does not require pro-ATG8 cleavage due to an already exposed C-terminal glycine [110]. In *Plasmodium falciparum*, it seems only one ATG8 isoform exists, which also does not require pre-processing for the same reason. Thus, such cells have the potential to be a tool to identify endogenous ATG8ylated proteins. It has not yet been demonstrated that protein ATG8ylation exists also in plant and Plasmodium cells, but given the conservation of the pathway, it is tempting to presume it does.

A further method to potentially identify ATG8ylated proteins could rely on the differences in the conjugation enzymes between membrane ATG8ylation and protein ATG8ylation. Since protein ATG8ylation does not require ATG5 in cells [104], one could generate ATG5-deficient cell lines that would be defective in canonical membrane ATG8ylation, yet still capable of protein ATG8ylation. A differential proteomic or high-content imaging approach may be able to determine novel ATG8ylated proteins in such a setting. However, this may be complicated by the other compartments that can be attached with ATG8 in an ATG5-independent manner. Furthermore, it has recently been shown that in the absence of ATG5, an ATG3–ATG12 conjugate is formed that is required for secretory mechanisms [111]. Also, it has recently been proposed that multiple additional E3-like enzymes may exist to mediate selective ATG8ylation [112]. Further proof for a yet unknown E3 ligase required for protein ATG8ylation comes from a recent observation that reconstitution of protein ATG8ylation in *E. coli* by expression of ATG3 and ATG7 is not sufficient to produce ATG8ylated proteins [105] suggesting the lack of a crucial component such as an E3-like enzyme. The identification of such an E3-like ligase for protein ATG8ylation is a priority in future investigations.

Another possibility to identify ATG8ylated proteins is to explore published datasets. Several studies have aimed to reveal the network of ATG8 protein complexes. Overall, these could identify components of the organelles that are coated with ATG8, direct interaction partners of ATG8 via a LIR or other domain, and ultimately, the direct conjugates of ATG8 to proteins. Buried within these sets of binders and interactors could be proteins that are directly linked to ATG8 family members.

Many questions remain unanswered, such as what is the functional relevance of protein ATG8ylation? It has been suggested that protein ATG8ylation is a default process that occurs at basal levels and the main function of ATG4 proteins is thus to prevent aberrant ATG8ylation on target proteins. Also, it has been brought forward that ATG8ylation in general is a way for cells to cope with different forms of stress [113]. Another possibility is that ATG8ylation has an important function as novel post-translational modification in itself, which remains to be studied in more detail.

A recent study identified an increase in the abundance of ATG8ylated proteins in a model of amyloid lateral sclerosis, suggesting that protein ATG8ylation is linked to neurodegenerative disease [114]. Others have suggested a role in stress responses or oxidative stress [3,112]. It remains to be seen whether protein ATG8ylation only occurs under stress or in disease contexts or whether it has a physiological role. The identification of more ATG8ylated proteins will provide meaningful steps toward understanding the significance of this post-translational modification.

## Data Availability

The authors confirm that the data supporting the findings of this study are available within the article.
